# Artificial cell mimics as simplified models for the study of cell biology

**DOI:** 10.1177/1535370217711441

**Published:** 2017-06-04

**Authors:** Ali Salehi-Reyhani, Oscar Ces, Yuval Elani

**Affiliations:** Department of Chemistry, Imperial College London, London SW7 2AZ, UK

**Keywords:** Artificial cells, biomimetics, synthetic biology, biophysics

## Abstract

Living cells are hugely complex chemical systems composed of a milieu of distinct chemical species (including DNA, proteins, lipids, and metabolites) interconnected with one another through a vast web of interactions: this complexity renders the study of cell biology in a quantitative and systematic manner a difficult task. There has been an increasing drive towards the utilization of artificial cells as cell mimics to alleviate this, a development that has been aided by recent advances in artificial cell construction. Cell mimics are simplified cell-like structures, composed from the bottom-up with precisely defined and tunable compositions. They allow specific facets of cell biology to be studied in isolation, in a simplified environment where control of variables can be achieved without interference from a living and responsive cell. This mini-review outlines the core principles of this approach and surveys recent key investigations that use cell mimics to address a wide range of biological questions. It will also place the field in the context of emerging trends, discuss the associated limitations, and outline future directions of the field.

**Impact statement:**

Recent years have seen an increasing drive to construct cell mimics and use them as simplified experimental models to replicate and understand biological phenomena in a well-defined and controlled system. By summarizing the advances in this burgeoning field, and using case studies as a basis for discussion on the limitations and future directions of this approach, it is hoped that this minireview will spur others in the experimental biology community to use artificial cells as simplified models with which to probe biological systems.

## Introduction

The construction of artificial cells that resemble biological cells in form and function is a rapidly growing area of research. The versatility of artificial cells as a platform has led to many potential applications, for example as engineered micromachines that can sense their environments, perform user-defined tasks, move autonomously, and undertake chemical synthesis.^1–6^ Vesicle-based artificial cells encapsulating biomolecules have been shown to be capable of hosting ubiquitous cellular reactions^7^ including polymerase chain reaction,^8^ RNA polymerization,^9^ protein synthesis,^10^ and coupled transcription and translation.^11^ A related application is their use as simplified mimics of real biological cells. In this way fundamental questions relating to cell biology, biochemistry, molecular biology, and systems biology can be addressed through an 'understanding by building' approach. This application is the focus of this mini-review.

This discipline falls within the field of synthetic biology, which deals with the design of biological modules, systems, and machines using engineering principles. We focus on the bottom-up approach to the field; here building blocks—either synthetic or biological—are used to construct cell mimics with cell-like features and behaviors. This is in contrast to the top-down approach where cells are altered or re-designed using genetic engineering techniques. As cells engineered from the top-down are living to begin with, their complexity approaches that of non-engineered cells. On the one hand this is advantageous: the cells very closely approximate non-engineered cells and already possess intricate metabolic pathways that can hijacked and repurposed for a range of biotechnological applications (e.g. as sensors and as living reactors for chemical synthesis). The tools and technologies to manipulate these cells are well-developed and from a technological standpoint it is simpler to re-engineer an organism than to construct one from scratch. This field is thus more advanced than that of bottom-up synthetic biology. However, as is described in the sections below, the complexity of top-down modified cells also hinders their use as cell models. There have been extensive reviews on the use of these cells as cell models (specifically minimal cells), and we refer the reader to these.^12–15^

**Figure 1:**
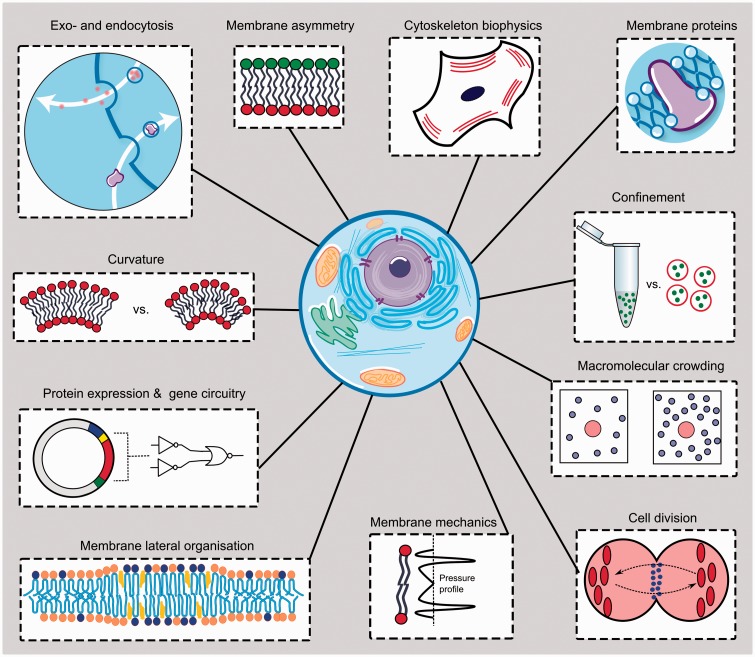
Schematic summarizing some of the cellular components and biological phenomena that have been studied using artificial cell mimics

The requisite complexity of an artificial cell model depends on the question at hand. While a spectrum of different artificial cell architectures exist—including droplets,^16^ coacervates,^17^ vesicles,^18^ polymersomes,^19^ and proteinosomes^20^—they have only recently started to achieve the complexity needed for them to adequately imitate various facets of cell biology. This mini-review will summarize the benefits of using artificial cell models and will examine the most significant examples of their use in addressing biological questions. This includes investigations relating to cellular membranes, the mechanisms of action of various cellular structural components, the effects of molecular crowding, and the design of gene circuits. A summary of the investigations covered is shown in Figure 1. We will use these case studies as a basis for discussion and highlight some of the limitations of the approach.

## The artificial cell advantage

Eduard Buchner’s discovery of cell-free fermentation by enzymes^21^ demonstrated that not all chemical transformations in biological systems requires organized, living cells. Bottom-up synthetic biology, then, is very much a modern biochemical application of this principle.

The molecular composition of cells is extremely varied and creating a full molecular census of a mammalian cell—metabolites, DNA, RNA, proteins, and lipids, all of which can exist in a variety of chemically modified states—remains a pipe dream. Consideration of the intricate complexity of the proteome exemplifies the enormity of the challenge which systems biology faces. There are of the order of 20,000 protein coding genes in human DNA^22^ which are estimated to produce, including splice variants and post-translational modifications, approximately 1 × 10^6^ different types of proteins.^23^ While estimates do vary and this number is often revised with each advancing study, it is clear that each protein species does not exist in isolation but interacts with other proteins and also other biomolecules; thus a complex web of interactions and signaling pathways exists within a cell. The molecular parameter space of cells is therefore huge, and this presents an inherent challenge in disentangling information associated with each component, especially when attempting to do so in a quantitative manner. Within such highly interconnected chemical networks, there is significant potential for redundancy as there are a number of alternative pathways linking each part of the network with the others. As a result, off-target effects can make isolating components in a systematic manner extremely difficult.

Herein lies the key attraction of using artificial cells as models of their biological counterparts, of which the advantages outlined below hang—simplicity. These models are simple enough to experimentally derive a fundamental understanding of the underlying mechanisms of cell biology, and yet can still approximate aspects of biological processes only emergent in more complex systems.

Artificial cells can be generated using a variety of approaches including bulk self-assembly and emulsion-based methods.^24,25^ There has been an increasing interest in using microfluidics as an enabling technology. This has allowed artificial cells to be constructed with increasing levels of control over size, architecture, degree of compartmentalization, connectivity between compartments, membrane composition and molecular organization, and content of internal volumes—a versatility that underpins many of the advances in this area.^18,26–29^ Given such control, the prospect of synthesizing cells with systematically altered variables in a controlled manner can be realized.

Constructing a simplified cell, for example with only one pathway linking two system components together, will allow a comprehensive understanding of this pathway to be achieved. Furthermore, artificial cells are not living systems and do not necessarily require culture conditions or nutrients as would a biological cell, nor do they respond to stress stimuli, unless engineered to do so. Thus, artificial cells may be interfaced with a range of probing molecular tools (nano- and micro-particles, reactive chemical species, etc.) which may be incompatible with biological cells. Finally, the construction of simplified experimental physical models also facilitates the superposition of descriptive mathematical models and molecular simulations, specifically multi-scale modeling that captures biological information across spatial, temporal, and functional scales. It should be noted this is not the focus of this article, and we refer the reader to reviews on this subject.^30–32^

**Figure 2:**
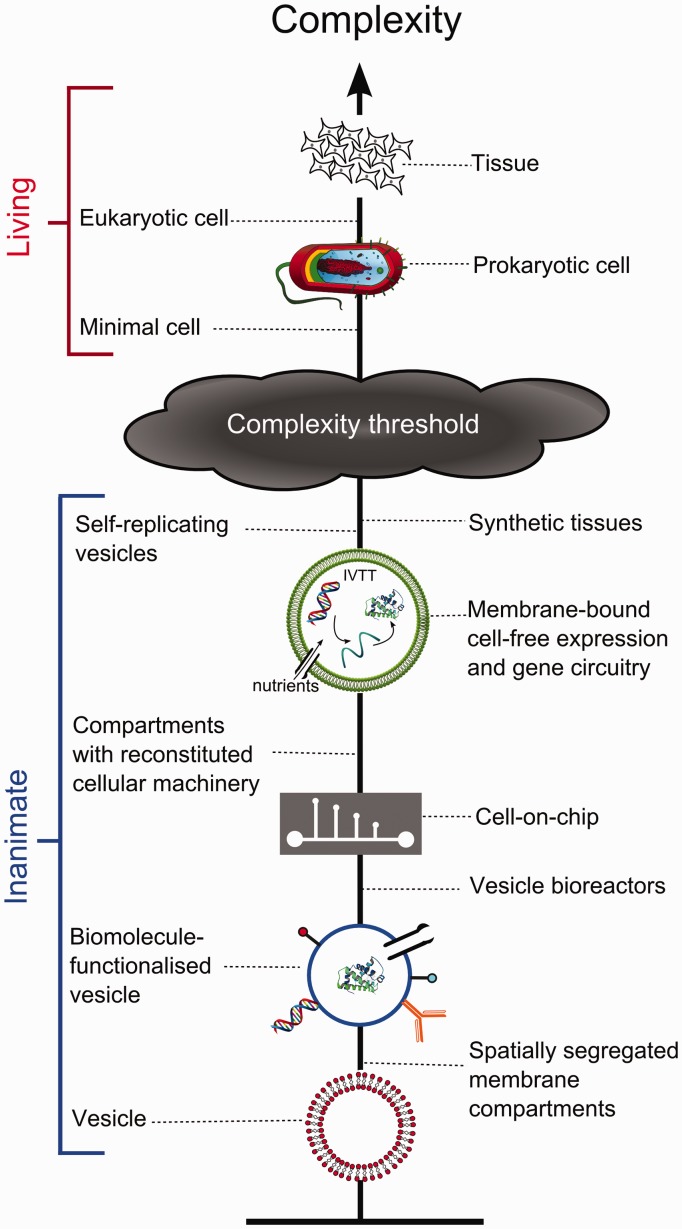
Schematic demonstrating the increasing complexity of various cell-mimetic structures. As they get more cell-like a threshold will be reached where they can be considered living. It should be noted that although the precise location of this threshold is by definition an arbitrary one, artificial cells constructed from the bottom-up are currently still a considerable distance away from this point

The above points demonstrate the potential advantages of using artificial cell models. However, for these to be realized, the construction of artificial cells must reach a stage where they can adequately represent real cells, which can be achieved through the introduction of an increasing number of components (Figure 2).

## Cell membrane mimics

Historically, the plasma membrane has been the most well-developed cellular component to be mimicked and used as a model. This is in part due to the relative simplicity of synthesizing lipids and their innate property of spontaneous self-assembly into membranous structures. Most often plasma membrane mimics take the form of lipid vesicles, though other structures exist as well, for example black lipid membranes, supported lipid bilayers, and nanodiscs.^33^ Various aspects of model membranes can be tuned for the systematic study of their effects on biological processes, including lipid composition,^34–37^ membrane asymmetry,^35^ degree of confinement and compartmentalization,^38^ as well as their intrinsic mechanical properties.^39,40^

The development of droplet microfluidic technologies for vesicle construction has greatly enhanced the degree of control of these variables. For example, it has enabled the routine fabrication of asymmetric vesicles^41^ which have a compositional variation in lipid species present in two leaflets of the bilayer. This had led to studies showing the effect of asymmetry on the global biophysical property of bending rigidity,^41–43^ which describes the energy required to bend a membrane and has been suggested to influence protein folding and gating.^35,44^ Membrane asymmetry is an almost universal feature of biological membranes, yet a precise understanding of its effects is currently poorly understood. It is hoped that advances in accurately constructing physical models mimicking this feature will help address this.

Membrane mimics have also been used to investigate the phenomena of lateral membrane organization and segregation, often referred to as lipid rafts.^45^ These are believed to be compartmentalized surfaces on cellular membranes that are more tightly packed and ordered than the surrounding bilayer. Lipid rafts have been suggested to facilitate various cellular processes such as the assembly of signaling molecules, coordination of membrane protein trafficking, modulation of membrane fluidity, and acting as platforms for virus entry.^46^ Analogues of lipid rafts can be engineered into lipid vesicles. In the simplest case, membranes composed of lipid mixtures, for example consisting of low and high melting point lipids and cholesterol, can display phase coexistence.^47^ Such membranes exhibit defined domains with different degrees of molecular order (for example, gel, liquid-ordered, and liquid-disordered).^48^ These have been used to shed light on the response of raft-like domains to temperature and pressure^47,49^ and on the aggregation/recruitment of biomolecules at defined points on the membrane surface.^50–52^ It should be noted that the analogy between cellular lipid rafts and domains found in model membranes has been disputed, which effectively demonstrates the care which one must take when adopting simplified systems as biological models. Membrane rafts are transient structures ranging in size from tens to hundreds of nanometers, with nanosecond lifetimes, and which do not exhibit line tension, whereas membrane domains are micrometer-sized structures which will coalesce over time.

The ability to systematically vary membrane lipid composition has enabled investigation into the effect of membrane biophysical parameters on the gating, folding and insertion of embedded, and peripheral membrane proteins.^53^ Examples include investigations into protein domain folding,^54^ the effect on intra-bilayer pressure profiles on the gating of mechanosensitive channels,^35^ the role of membrane tension in skin sensory transduction,^55^ the behavior of embedded proteins as stretch-activated osmotic release valves,^56^ and the potential role of stored curvature elastic stress in maintaining lipid homeostasis via a negative feedback loop.^40^ Finally, cell-mimetic vesicles have proved to be a powerful system on which to study the effects of geometric membrane curvature on protein binding, activity, and function,^57,58^ as well as protein-induced membrane deformation that is implicated in trafficking^59^ and lipid and protein distribution.^60^

## Investigating cellular structural components

Artificial cell models are increasingly used in investigations relating to the mechanisms of action of various structural components and associated machineries. One research avenue which has proved particularly fruitful is the use of cell mimics for the study of cytoskeleton biophysics.

Membrane-bound cell mimics were used to quantify the forces involved in actin polymerization, as well as spatially resolving the forces exerted in different membrane regions.^61^ This revealed that the existence of retractive or propulsive forces depends on local membrane curvature, and allowed for the measurement of the strength of binding between membranes and the actin gel. Others have studied interactions between myofilaments and membrane-bound actin filaments through reconstitution of a minimal actin cortex, leading to a proposed mechanism of tension and compressive stress build up that is involved in myofilament-induced actin fragmentation.^62^ There have also been extensive studies utilizing cell mimics to dissect the elementary biochemical processes that govern cytoskeleton-based morphology changes and mechanical force generation.^63^ In a recent study, Maeda *et al*. demonstrated cell-free synthesis of MreB filaments (a homologue of actin) inside lipid vesicles.^64^ These filaments successfully assembled into micron-sized rigid bundles which adhered to and deformed the vesicle surface. Cellular and membrane deformations can also be the result of molecular motors exerting a force on membranes by interacting with their cytoskeleton, and cell mimics have proved crucial in a quantitatively assessing the processes involved.^65^ Processes such as these that involve deformations in synthetic membranes have also been key to understanding the biophysical basis for endocytosis and exocytosis;^66^ for example through BAR domain proteins and clathrin-mediated process,^67,68^ and through the action of viruses^69^ and bacterial toxins.^70,71^

**Figure 3:**
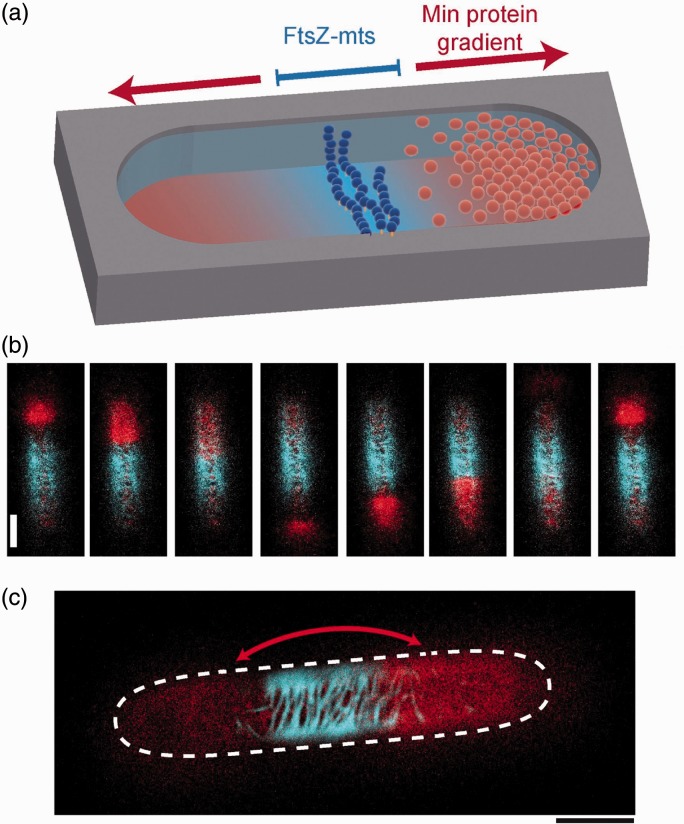
Min protein oscillation and FtsZ accumulation in a cell-like compartment. (a) Schematic of pole-to-pole Min proteins oscillation along the long axis of a compartment, and accumulation of FtsZ-mts along the equator when Min proteins concentration is lowest. (b) Time course and (c) superimposed image of the location of the oscillating Min system (red) and FtsZ-mts (blue) when reconstituted together. Image modified from referenced publication;^109^ Reconstitution of self-organizing protein gradients as spatial cues in cell-free systems. Published and distributed under the terms of the Creative Commons Attribution License

**Figure 4:**
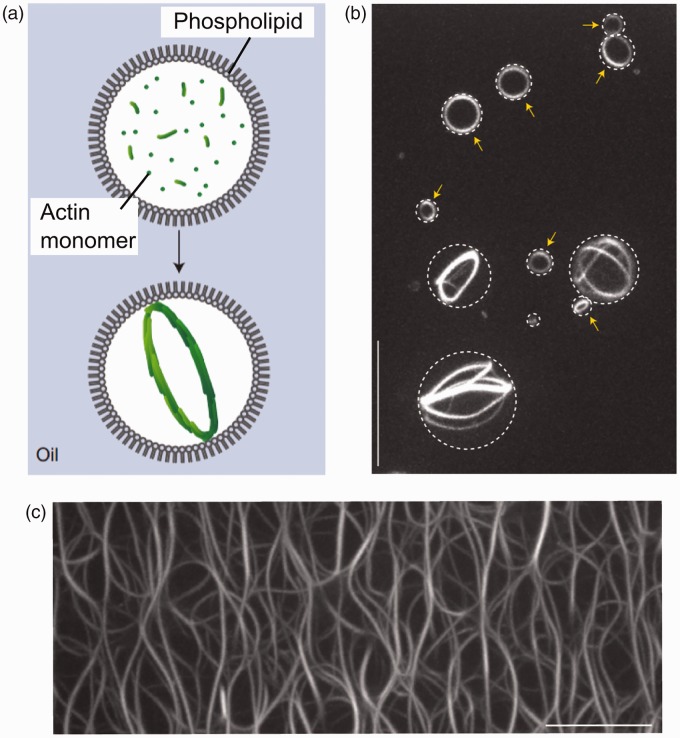
Self-organization of actin networks through confinement in a biomimetic compartment. (a) Schematic of experimental model of a lipid-coated cell-sized droplet with encapsulated actin monomer. (b) Self assembled actin networks form at the droplet equator. (c) This is in contrast to an unconfined environment when an unordered network assembled. Reprinted by permission from Macmillan Publishers Ltd.,^73^ copyright (2015)

There has been a drive at using biomimetic cells as models to understand the process of cell division (cytokinesis), a process which is composed of several constituent parts. The first is identification of a defined spatial location where membrane fission will occur through the accumulation of accessory proteins that form circular structures that can contract and define a division site,^72,73^ followed by membrane abscission.^74,75^ The Schwille group devised an *in vitro* system comprised of the bacterial Min system which enables the establishment of an intrinsically defined protein gradient.^76^ In cells, this operates by positioning the cell-division machinery at defined locations though pole-to-pole oscillations (Figure 3). This was reconstructed in cell-mimetic lipid-coated droplets proving to be a simple platform for investigations of cell-division proteins, with particularly revealing results regarding the dependence of the morphology of protein bundles on compartment size.^77^ Others have shown that actin polymerization within cell-mimetic droplets induced the spontaneous formation of single ring-shaped actin bundles^73^ (Figure 4) which can be viewed as a precursor to the splitting the cell body in two.

Although the full process of cellular division has not yet been fully recapitulated inside a synthetic system, the potential of such systems cannot be overstated. In future, synthetic cells may be used not only for understanding such critical biological process, but also for the construction of a truly artificial cell capable of reproduction and eventually evolution.^78^

## Macromolecular crowding

It has long been known that protein folding, stability, and function, as well as enzymatic reaction kinetics and mechanisms, are influenced by molecular crowding through reduced diffusion times and increased molecular binding rates.^79–82^ The construction of cell mimics has allowed the effects of confinement and compartmentalization to be studied in a controlled environment. This is critical as up to 40% of the cellular environment is occupied by macromolecules.^83^ As a demonstration of this importance, Tan *et al*. have shown that macromolecular crowding increased the robustness of gene expression, and that expression kinetics could be modulated using parameters such as compartment volume, and the size of crowding agents.^84^ Others have suggested that the very process of entrapment of a large number of macromolecules inside micron-sized compartments is strongly dependent on spontaneous protein crowding effects.^85^

## Gene circuits

One powerful approach for the construction and study of biological systems in a non-cellular, non-living system is *in vitro* transcription and translation (IVTT). In comparison to *in vivo* protein synthesis, the concentration of relevant biomolecules (including genes, ribosomes, and polymerases) can be systematically varied, all parameters (including ATP concentration and redox states) can be known and controlled, reporter response is quantitative, and there is no need for time-consuming cellular transformations; this allows for a large parameter space to be studied.

As a consequence this has mainly been used as tools for genetic circuit design,^86^ to increase our mechanistic understanding of the principles involved from an engineering perspective, to investigate informational and metabolic processes *in vitro*, and for rapid prototyping of biochemical networks before incorporation into living cellular chassis.^87,88^

Although such studies were conducted with bioengineering applications in mind and not for fundamental science, they clearly demonstrate how the line between the two can become blurred. The more complex the circuits become, the more representative they are of biological systems. In this vein, increasingly complex analogues of natural circuits have been constructed using *in vitro* systems.^89^ These include multi step cascades where the protein product of one stage is used to activate or repress the next stage,^86^ two stage genetic networks,^90^ oscillations,^91,92^ bistable circuits using transcriptional switches,^93^ simple logic gates, and positive and negative feedback loops.^94^ IVTT has also been used for the assembly of various protein complexes, including ATP synthase,^95^ ribosomes,^96^ and synthetic protein nanoassemblies.^97^ Indeed, the entire cascade needed for viral assembly, including genome replication, synthesis, and viral particle assembly, has been achieved from a cell-free mixture.^98^

IVTT is mostly conducted in non-confined bulk format. However there are an increasing number of studies that compartmentalize reactions in space in order to mimic the effects of confinement. Weitz *et al*. have shown that compartmentalization in to fl-pl droplets result in large variation in protein expression behavior due to stochastic partitioning of low-concentration species.^99^ The initial variability in starting conditions led to a diversity in dynamic behaviors of the system (in this case fluorescence oscillations), and as predicted, due to the small number effect, this variability was larger with smaller droplets.

## Cells-on-chip

Construction of artificial cells for fundamental biology need not be limited to membrane-encapsulated systems. There have been several recent examples at using the confines of a microfluidic device itself to define the boundaries of a synthetic cell, allowing diffusion of nutrients and products into and out of the “cell” in precisely defined and controlled rates.

In one recent example, functionalization of the silicon substrate of a microfluidic device with a protein-coding DNA brush was achieved, with the resulting chip-based cell being capable of metabolism, protein synthesis, and inter-compartment communication.^100^ In this system, channel geometry was used to simulate parameters such as protein degradation, allowing for precise manipulation of the system to produce dynamic patterns including protein expression oscillations, which were modeled on the basis of simple equations.

Others have assembled microfluidic devices capable of cell-free expression that could be controlled using valves, to enable high-throughput generation and analysis of protein-interaction networks,^101^ revealing previously undescribed interactions. Such platforms have significant potential for biological investigations, as they provide a further level of user-control compared to membrane-encapsulated lipid vesicles, and offer a means to study biological networks outside the confines of a living cell.

## Perspectives and conclusions

The synopsis above shows that cell mimics have been used for a range of studies in fundamental biological research, and towards the construction of functional artificial cells. However, there are still several areas where the full potential of such systems has yet to be exploited. One is their use as standards for single-cell analysis, where the precisely defined chemical and biochemical composition of artificial cells makes them particularly attractive as tools to calibrate new technologies, for example those depending on immunofluorescence and fluorescence-activated cell sorting.^102,103^ In addition, unlike biological cells, their reduced complexity puts them within reach of computational methods. Artificial cells are thus appealing experimental models to complement simulations. This is a research area which has only recently begun to be explored, for example to simulate a cell-free expression systems composed of purified components.^104^ The process of assembling cell mimics for fundamental research will also lead to improved and better-understood engineering rules for their construction, which will in turn feed into other applications, for example in the use of artificial cells as smart drug and gene delivery vehicles that contain functional biological machinery.

Despite the potential of cell-mimics in fundamental research, an assessment of their limitations is necessary, in part to identify necessary future research directions to combat these, but also to recognize what applications are less suited to their use. Their primary limitation is that associated with all models, namely that their vastly reduced complexity by definition means they can only roughly approximate biological cells, and investigations must be complemented by *in vivo* studies. This also renders cell mimics more useful in the study of isolated cellular processes, and not in investigating emergent cellular events comprised of a multitude of systems operating in concert. Furthermore, living cells do not exist in isolation, but instead are part of a matrix that affects their behavior and function, an aspect which is yet to be adequately addressed in minimal systems. Similarly, in the medium-term at least, although a cell-level organization can be mimicked, higher order structures such as tissues are within further reach, although organ-on-chip^105,106^ and related^107,108^ technologies may prove useful in this regard. A final major obstacle to the field which has yet to be addressed is the great difficulty in measuring or monitoring the exact composition of an artificial cell once it has been synthesized.

In short, a gulf of complexity exists between today’s artificial cells and that of even the simplest of organisms. It is not clear how long it will take to achieve a self-sustaining, living artificial cell. If the rate of advancements in the reduced cost of gene sequencing are any indication of the pace of wider biotechnological progress, then the opportunities for biology and medicine that artificial cells offer may be upon us in no time at all.

The forces which have facilitated the rapid rise of this research area—namely the reduced cost of DNA synthesis, advances in robust biomolecular extraction and reconstitution protocols, availability of commercially available cell-free expression kits, novel vesicle generation strategies, and the development of integrated microfluidic devices allowing controlled construction of synthetic cells—will continue to allow for the expansion of the frontiers of the use of cell mimics for fundamental research. This will likely be aided by several emerging technologies. These include 3D printing for rapid prototyping of microfluidic devices (also helping to democratize the discipline), additive manufacturing of biological systems, and the development of novel genetic engineering technologies (e.g. CRISPR). Integrating various facets of cell mimicry which have previously been examined in isolation will also be key for the construction of multi-component systems that more closely approximate biological cells. Focus on multi-disciplinary research will also be invaluable for the advancement of these technologies.
